# Therapeutic Approaches for Peripheral and Central Neuropathic Pain

**DOI:** 10.1155/2019/8685954

**Published:** 2019-11-21

**Authors:** Délia Szok, János Tajti, Aliz Nyári, László Vécsei

**Affiliations:** ^1^Department of Neurology, Interdisciplinary Excellence Centre, Faculty of Medicine, University of Szeged, Semmelweis u. 6, H-6725 Szeged, Hungary; ^2^MTA-SZTE Neuroscience Research Group, Semmelweis u. 6, H-6725 Szeged, Hungary

## Abstract

Neuropathic pain is a chronic secondary pain condition, which is a consequence of peripheral or central nervous (somatosensory) system lesions or diseases. It is a devastating condition, which affects around 7% of the general population. Numerous etiological factors contribute to the development of chronic neuropathic pain. It can originate from the peripheral part of the nervous system such as in the case of trigeminal or postherpetic neuralgia, peripheral nerve injury, painful polyneuropathies, or radiculopathies. Central chronic neuropathic pain can develop as a result of spinal cord or brain injury, stroke, or multiple sclerosis. As first-line pharmacological treatment options, tricyclic antidepressants, serotonin-norepinephrine reuptake inhibitors, and gabapentinoids are recommended. In trigeminal neuralgia, carbamazepine and oxcarbazepine are the first-choice drugs. In drug-refractory cases, interventional, physical, and psychological therapies are available. This review was structured based on a PubMed search of papers published in the field from 2010 until May 2019.

## 1. Introduction

The current definition of neuropathic pain (NP) was released almost one decade ago by the International Association for the Study of Pain (IASP) [1]. Based on this statement, NP is caused by a lesion or disease of the somatosensory (peripheral and/or central) nervous system. This special type of pain affects some 7-10% of the general population globally, predominantly in patients above 50 years of age [2]. The characteristics of NP are clearly distinct from those of nociceptive pain, which together represent the two fundamental groups of pain conditions. However, according to a new mixed pain concept, an additional group of pain disorders is proposed, which is referred to as “nociplastic pain” [3]. Chronic NP includes peripheral and central NP conditions [4].

A substantial advancement in this field is the latest classification of these heterogeneous pain syndromes, published by the IASP in 2019 [5]. The subtypes of chronic peripheral NP are the following: trigeminal neuralgia (TN), chronic NP after peripheral nerve injury, painful polyneuropathy, postherpetic neuralgia, and painful radiculopathy. The following forms belong to chronic central NP: chronic central NP associated with spinal cord injury (SCI), chronic central NP associated with brain injury, chronic central poststroke pain, and chronic central NP associated with multiple sclerosis (MS) [4] (Tables 1 and 2). In general, NP conditions are underrecognized, underdiagnosed, and undertreated.

Treating NP is a real challenge for physicians. The management of NP targets predominantly the clinical symptoms instead of the causative factors. Currently available treatment options include both pharmacological and nonpharmacological approaches.

Regarding pharmacological therapies in NP, tricyclic antidepressants (TCA; e.g., amitriptyline), serotonin-norepinephrine reuptake inhibitors (SNRIs; i.e., duloxetine and venlafaxine), and gabapentinoids (i.e., gabapentin and pregabalin) are recommended as first-line treatments. In second-line, weak opioid analgesics (e.g., tramadol and tapentadol) are recommended. Topical agents (i.e., lidocaine plaster and capsaicin patch) are recommended as second-line pharmacological treatments exclusively in peripheral NP. As third-line drugs, strong opioids (e.g., morphine and oxycodone) are recommended both in central and peripheral NP conditions, whereas botulinum toxin type A-haemagglutinin complex (BoNTA) can be recommended only in peripheral NP conditions. In TN, carbamazepine (CBZ) and oxcarbazepine (OXC) are the first-choice drugs.

Nonpharmacological therapeutic options for drug-refractory NP include the following approaches: interventional therapies (e.g., peripheral nerve blockade, epidural steroid injection, sympathetic nerve/ganglion treatment, intrathecal drug/medication delivery, and peripheral and central neurostimulation), physical therapies (e.g., massage, ultrasound, transcutaneous electrical nerve stimulation (TENS), laser, and mirror therapy exercise training), and psychological therapies (cognitive behavioural therapy (CBT), psychotherapy, and internet-delivered psychological therapies).

Papers selected for this work were searched by PubMed with the keywords: “peripheral neuropathic pain”, “central neuropathic pain”, “trigeminal neuralgia”, “chronic neuropathic pain after peripheral nerve injury”, “painful polyneuropathy”, “postherpetic neuralgia”, “painful radiculopathy”, “chronic central neuropathic pain associated with spinal cord injury”, “chronic central neuropathic pain associated with brain injury”, “chronic central post-stroke pain”, or “chronic central neuropathic pain associated with multiple sclerosis” and “therapy”, “treatment”, “pharmacological”, “non-pharmacological”, “investigational therapy”, “physical therapy”, or “psychological therapy”. Only abstracts published in English were considered. The PubMed search was done for papers published from 2010 until May 2019.

The aim of this review was to provide an expert view summarizing the current status of available therapeutic possibilities both in peripheral and central NP conditions, based on the novel IASP classification system of chronic NP conditions. An additional goal was to present the results of the clinical trials of nonpharmacological approaches in different types of drug-refractory NP.

## 2. Chronic Peripheral NP

### 2.1. Trigeminal Neuralgia

According to the new concept of classification of chronic pain by the IASP, TN is classified as a subclass of “chronic peripheral NP”; however, it has “chronic secondary headaches and orofacial pains” as an additional parent [4].

The definition of TN is based on the diagnostic criteria of the latest classification of the International Headache Society (ICHD-3) [6, 7]. This is a devastating pain condition characterized by recurrent unilateral orofacial ache restricted to one or more branches of the trigeminal nerve. The characteristics of the pain can be described as electric shock-like, shooting, stabbing, or sharp in quality and severe in intensity. The painful attacks last from a couple of seconds to a maximum 2 minutes. This painful paroxysm can be triggered by innocuous mechanical stimuli or orofacial movements. Even more, in some cases, involuntary painful contractions of the muscles on the face can occur, as it was referred to by the previous term “tic douloureux.”

TN is a subtype of painful cranial neuropathies and is divided to classical, secondary, and idiopathic forms. Painful trigeminal neuropathy is classified as a different entity [6–8]. The essence of classical TN is that the patients have microvascular compression with morphological changes (nerve atrophy or displacement) of the trigeminal nerve root entry in the pons, as demonstrated by high-resolution 3T MRI [6, 7]. This subtype of TN represents some 80% of the all TN patients [9]. Secondary TN can be caused by various neurological disorders, such as tumours in the cerebellopontine angle, MS (i.e., demyelinating lesion in the pons), or an arteriovenous malformation. Clinically, the main difference between the classical and the secondary TN is that secondary TN presents with sensory abnormalities in the orofacial area innervated by the trigeminal nerve [6, 7, 10]. The last category of TN is idiopathic, which means unknown cause (i.e., proper diagnostic work-up does not confirm any lesion or disease as causative) [6, 7]. Idiopathic TN makes up about 11% of all TN cases [7].

The incidence of TN is from 4.3 to 27 cases per 100,000 capita per year and is more common in persons older than 60 years. Regarding the sex, TN is more frequent in women (5.9/100,000 cases per year) than in men (3.4/100,000 cases per year) [9].

The diagnosis of TN requires proper medical history. As regards neurological physical examination, it should be underlined that this type of pain can be triggered and in the classical form, sensory disturbances are usually absent. As for instrumental investigation, the fundamental method is high-resolution 3T MRI, which gives us information about the status of the posterior cranial fossa. The differential diagnosis of TN includes other cranial neuralgias (e.g., glossopharyngeal neuralgia), other facial pains (e.g., persistent idiopathic facial pain), primary headache disorders (such as cluster headache or other trigeminal autonomic cephalalgias), and odontogenic diseases such as cracked tooth, caries, or pulpitis [11].

Treatment options of TN can be divided into a pharmacological and a surgical part. Pharmacological therapy includes CBZ, OXC, lamotrigine, pregabalin, gabapentin, baclofen, or BoNTA injection [8, 11]. Pharmacological treatment recommendations are similar in the classical and secondary forms of TN. Among these pharmacons, CBZ and OXC (as sodium ion channel blocker antiepileptics) as gold standards have strong recommendations as first-line and long-term treatment for TN [8, 11, 12]. The recommended daily dose is 200-400 mg for CBZ and 300-600 mg for OXC [13]. The majority (90%) of TN patients responds well to these drugs [7]. The number needed to treat (NNT) for CBZ is low (NNT = 1.7) [11]. Contrarily, the number needed to harm (NNH) for CBZ is high (NNT = 24 for severe side effects and NNT = 3.4 for minor side effects) [11]. The most common adverse events of CBZ are somnolence, dizziness, drowsiness, rash, liver damage, hyponatraemia, tremor, and ataxia [8, 11]. For TN patients who cannot tolerate the recommended full dose of CBZ or OXC, an add-on treatment with lamotrigine or baclofen can be advised [14]. BoNTA represents a third-line treatment option for treating TN [2, 8].

Surgical therapeutic possibilities include microvascular decompression (MVD), gamma knife radiosurgery, glycerol rhizolysis, internal neurolysis, and radiofrequency thermocoagulation. MVD is the first choice for drug-refractory TN patients with neurovascular contact. Around 73% of patients still report significant pain relief five years after MVD treatment [11].

### 2.2. Chronic NP after Peripheral Nerve Injury

This type of pain originates from peripheral nerve lesions and can be recurrent or persistent [4]. Based on the latest classification, “chronic NP after peripheral nerve injury” is a third-level diagnosis of the “chronic NP” group and can also be derived from “chronic posttraumatic pain,” an additional parent [4, 15]. In addition, many disorders listed in the “chronic postsurgical pain” category can be associated with neuropathic component.

#### 2.2.1. Chronic Postsurgical Pain Disorders with Neuropathic Component

Chronic postsurgical pain develops after a surgical procedure. Disorders related to this special pain type are the following: chronic pain after amputation, chronic pain after spinal surgery, chronic pain after thoracotomy, chronic pain after breast surgery, chronic pain after herniotomy, chronic pain after hysterectomy, and chronic pain after arthroplasty [15]. Most of these conditions associate with neuropathic components in about half of the patients, which is reflected also by their therapeutic options.

*(1) Chronic Pain after Amputation*. By definition, chronic pain after amputation means that the pain developed after surgical amputation of a body part (e.g., limb, breast, tongue, teeth, genitalia, eye, or rectum) [15]. The most common localization of chronic pain after amputation is the distal part of the amputated limb (i.e., stump or phantom limb pain). The prevalence of phantom limb pain is between 30 and 85% [16, 17]. Pharmacological treatment of phantom limb pain is still unresolved, based on a recent Cochrane meta-analysis [18]. The suggested pharmacons are *N*-methyl-D-aspartate (NMDA)-receptor antagonists (e.g., ketamine and memantine), gabapentinoids (i.e., gabapentin and pregabalin), TCAs (e.g., amitriptyline), and opioids [18, 19].

*(2) Chronic Pain after Spinal Surgery (Failed Back Surgery Syndrome (FBSS))*. The location of this type of pain is the site of the operation or it can radiate to the lower extremities with neuropathic component [15, 20]. This chronic NP develops in an average of 20% of patient who underwent lumbar spinal surgery [15]. From therapeutic perspective, nonsteroidal anti-inflammatory drugs (NSAIDs) have only limited effect; however, the efficacy of gabapentin or pregabalin has already been proved in this type of chronic pain [20]. CBT-based treatment has also been reported to decrease postsurgical pain intensity [21]. In addition, spinal cord stimulation, as a nonpharmacological treatment option, has shown beneficial results as well [7, 20].

*(3) Chronic Pain after Thoracotomy*. It is defined as a pain after surgical incision to the chest wall. The prevalence of this type of chronic pain is about 50% of postthoracotomy patients, whereas about one-third develops neuropathic component as well. Optimal preemptive analgesia may give a chance to reduce this high rate [22]. As pharmacological treatment, gabapentin and pregabalin have shown a beneficial effect. As nonpharmacological treatment, neuraxial blockade or continuous paravertebral or epidural catheter can also be used [22].

*(4) Chronic Pain after Breast Surgery*. Surgical procedures in the breast area lead to the development of chronic pain in some 25-60% of the cases [15]. From pharmacological point of view, amitriptyline, gabapentinoids, venlafaxine, and topical capsaicin cream, whereas as a surgical method, autologous fat grafting have shown a significant effect in pain alleviation [23, 24].

*(5) Chronic Pain after Herniotomy*. This type of chronic pain originates from the surgical repair of an inguinal or femoral hernia. Around 20-30% of the operated patients develop chronic pain, and some 80% of these cases suffer from neuropathic pain [15]. The treatment of postherniotomy chronic pain is not yet solved. Based on a systematic review, pulsed radiofrequency ablation as an invasive pain treatment technique can relieve chronic postherniotomy pain [25].

*(6) Chronic Pain after Hysterectomy*. It can occur after the surgical (open transabdominal, laparoscopic, or transvaginal) removal of the uterus and the annexes. This type of chronic pain affects 5-32% of the operated women, with neuropathic component being present in 5-50% of the cases [15, 26]. Underlying the relevance of this condition, it is estimated that 1 out of 9 women undergoes hysterectomy in the USA [27]. Proper acute pain management during/after hysterectomy may influence the development of this postsurgical chronic pain [28].

*(7) Chronic Pain after Arthroplasty*. Following surgical replacement of a knee or hip joint, chronic NP develops in some 27-38% of the operated patients [15]. Novel surgical techniques and adequate perioperative pain management give a chance to reduce the risk of the development chronic pain after knee arthroplasty [29]. A multimodal approach of perioperative pharmacological management includes the administration of NSAIDs, acetaminophen, corticosteroids, clonidine, ketamine, gabapentin, or pregabalin [29, 30].

#### 2.2.2. Chronic Posttraumatic Pain Disorders with Neuropathic Component

These types of pain disorders develop after traumatic or burn injury of tissues, in particular, chronic pain after burn injury, chronic pain after peripheral or central nervous system injury, whiplash injury-associated pain, and chronic pain after musculoskeletal injury. After polytrauma, the frequency of the development of chronic pain is 46-85% [15]. In the treatment of this painful condition, pregabalin has shown strong efficacy, according to the latest Cochrane Database conclusion [31].

*(1) Chronic Pain after Burn Injury*. The background of this pain condition is multicausal (heat, cold, electricity, chemical, friction, or radiation injuries). Its prevalence is around 18-52% [15]. Neither pharmacological nor nonpharmacological therapies are well established [32]. A recently published retrospective cohort study concluded that early gabapentin administration for chronic pain after burn injury did not significantly diminish the pain intensity [33]. In the treatment of complex regional pain syndrome (CRPS), a condition caused by scarring after burn injury, the administration of calcitonin, bisphosphonates, mirror visual feedback treatment, and sympathetic ganglion blockade can be recommended with high levels of evidence [34]. CRPS can also result from trauma of the extremities. There are two subtypes: CRPS I and II. In CRPS I, there is no peripheral nerve injury, whereas in CRPS II, peripheral nerve injury is required for the diagnosis [35].

*(2) Chronic Pain after Peripheral Nerve Injury*. In addition to be a subclass of “chronic posttraumatic pain” [15], a subgroup of this condition that is associated with NP, i.e., “chronic NP after peripheral nerve injury” is in fact the parent of all pain conditions that are associated with peripheral NP, due to the novel multiple parenting classification system [4]. TCAs were found efficient in the treatment of this subtype of chronic pain [36]. In a case series of patients with therapy-resistant brachial plexus lesion that leads to chronic posttraumatic NP, peripheral nerve stimulation was effective [37].

### 2.3. Painful Polyneuropathy

Painful polyneuropathy is one of the most common chronic NP conditions. It is a heterogeneous group of NP and can be divided into diabetic and nondiabetic groups (including nondiabetic metabolic, autoimmune, infective (especially due to Human Immunodeficiency Virus (HIV) infection), toxic, genetic, and drug-induced) [4].

Based on data from epidemiological studies, the prevalence of *painful diabetic polyneuropathy* (*PDP*) is variable, ranging from 14.1% to 65.3% [38]. Due to its high frequency, PDP was the prototype disorder for the development of anti-NP therapeutic strategies. The first-line drugs in PDP as recommended by different international therapeutic guidelines are the following: duloxetine, gabapentin, pregabalin, TCAs (amitriptyline), and venlafaxine ER (extended release) [12, 39, 40]. Cochrane meta-analyses concluded that gabapentin, pregabalin, and duloxetine are effective for pain relief in PDP [31, 41, 42]. The second-line recommended pharmacons are opioids [12] and capsaicin (8%) patch [2]. The high-concentration (8%) capsaicin patch provided better pain relief than that with a substantially lower concentration in PDP patients [31]. BoNTA injection is recommended as a third-line treatment in PDP [2]. As regards OXC, a Cochrane meta-analysis concluded that it had little evidence for effectiveness in PDP [43]. Similarly, a systematic analysis demonstrated that oxycodone had only very low-quality evidence to be effective in PDP [44].

As a nondiabetic painful polyneuropathy, the prevalence of *HIV-related painful neuropathy* is around 35% in HIV-positive patients and it is up to 50% of patients with Acquired Immune Deficiency Syndrome (AIDS) [17]. In the treatment of HIV-associated painful neuropathy, topical capsaicin (8%) patch is recommended as first-line pharmacological therapy [12]. A recent Cochrane review analysing the efficacy of pregabalin in HIV-related neuropathy revealed ineffectiveness [31]. The high-concentration (8%) capsaicin patch provided better pain relief than that with a substantially lower concentration in HIV-related painful neuropathy [45].

### 2.4. Postherpetic Neuralgia

Postherpetic neuralgia (PHN) is defined as a chronic pain lasting more than 3 months that developed secondary to varicella zoster virus infection. PHN involves the dermatomes innervated by the affected cranial nerve or spinal dorsal root ganglions [4]. PHN develops in about 10% of infected patients; however, the risk of development increases with age, with 20% of patients over the age of 65 and 30% of those over the age of 80 developing PHN after zoster [46]. As first-line treatment for PHN, gabapentin, pregabalin, TCAs, and lidocaine plaster are recommended [12]. A Cochrane meta-analysis concluded that gabapentin was effective for pain relief in PHN [41]. Regarding pregabalin, the Cochrane Database also confirmed its efficacy in PHN [31]. For second- or third-line treatment options, capsaicin (8%) patch or opioids are recommended [12]. The high-concentration (8%) capsaicin patch provided better pain relief than that with a substantially lower concentration of capsaicin in PHN patients [45]. A systematic meta-analysis demonstrated that oxycodone, as a strong opioid, had only very low-quality evidence to be useful in providing pain relief in PHN [44]. BoNTA injection is recommended as a third-line treatment in PHN [2].

### 2.5. Painful Radiculopathy

By definition, painful radiculopathy is a pain that originates from a lesion or disease of the cervical, thoracic, lumbar, or sacral nerve roots [4].

A large clinical study revealed that the majority (54.7%) of patients suffering from chronic low back pain with radiculopathy have neuropathic component [47]. Other authors in Western European countries estimated this ratio to be somewhere between 20% and 35% [48, 49]. For patients suffering from painful cervical or lumbar radiculopathy with neuropathic component, pregabalin has been shown to be effective by certain studies [47, 50]. However, a recent randomized controlled trial (RCT) investigating the effect of pregabalin in patients with acute and chronic sciatica reported that pregabalin had no benefit [51]. Based on a literature review, gabapentin and nortriptyline diminished the intensity of cervical or lumbar radicular chronic pain [52]. A recent comprehensive review suggested TCAs and SNRIs (e.g., duloxetine and venlafaxine) for treatment of chronic low back pain with neuropathic component [53]. A Cochrane meta-analysis revealed that OXC in NP related to radiculopathy had little evidence for effectiveness [43].

## 3. Chronic Central NP

Chronic central NP is caused by a lesion or disease of the central somatosensory nervous system. It can be related to spinal cord or brain injury, stroke, or MS [4].

### 3.1. Chronic Central NP Associated with SCI

By definition, chronic central NP associated with SCI is caused by a lesion or disease of the somatosensory pathway in the spinal cord [4]. This condition can also be derived from the “chronic pain after spinal cord injury” category, referring to patients who have neuropathic component as well [15].

The prevalence of chronic pain after SCI is estimated to be between 40% and 70% [17]. Reflecting the high rate of patient with NP within this group, first-line pharmacological treatments recommended by the European therapeutic guideline for chronic pain after SCI include drugs with antineuropathic potential, such as gabapentin, pregabalin, and TCAs [12, 36]. A randomized, double-blind, placebo-controlled trial (*n* = 40 patients) revealed that BoNTA injection was effective in pain relief compared to placebo in intractable chronic NP in patients after SCI [54, 55]. Regarding nonpharmacological interventions, exercise programme led to mean reduction in pain intensity, whereas repetitive transcranial magnetic stimulation (rTMS), acupuncture, self-hypnosis, TENS, and CBT provided no evidence of efficacy in alleviating SCI-related chronic pain. Overall, based on the data of clinical trials, evidence regarding the efficacy of nonpharmacological treatments in chronic pain after SCI is not sufficient [56].

### 3.2. Chronic Central NP Associated with Brain Injury

By definition, chronic central NP associated with brain injury is caused by a lesion or disease of the somatosensory areas of the brain [4]. This pain condition can also be derived from “chronic pain after brain injury” category, referring to patients with neuropathic component [15].

The estimated global yearly incidence of traumatic brain injury (TBI) is 106/100,000 capita, whereas it is around 600/100,000 capita in the case of mild TBI [57, 58]. Some 13 million people is estimated to live with disabilities related to TBI in Europe and the USA [59]. The most common pain type related to mild TBI is headache, with a prevalence of 57.8%. Other frequent pain forms include neck or back pain and musculoskeletal pain [60].

Effective pain treatment after TBI may reduce the risk for pain chronification. For TBI-related chronic pain, topical agents, opioids (e.g., tramadol), anticonvulsants (gabapentin and pregabalin), and TCAs are recommended. Nerve blockades and epidural steroid administration may also be useful [61]. Patients with mild TBI who were treated with rTMS demonstrated significantly diminished pain intensity (as estimated by Numerical Rating Scale (NRS) and elevated physical and mental item scores of the Short-Form- (SF-) 36 questionnaire compared to the control group) [62]. Psychological distress and posttraumatic stress are often associated with TBI-related chronic pain. Different psychotherapeutic methods can be beneficial. In addition, long-term rehabilitation should be offered for these patients [63].

### 3.3. Chronic Central Poststroke Pain (CPSP)

Poststroke pain involves neuropathic and nociceptive mechanisms. The neuropathic mechanism occurs in patients who have thalamic or parietal lobe vascular lesion predominantly in the right hemisphere [4, 64]. The prevalence of CPSP is estimated to be between 8% and 30%, with a predominance in young stroke patients (being twice as frequent as in older patients) [17, 64, 65]. Typical features of CPSP are constant or intermittent pain, hyperalgesia, and allodynia [64, 66]. Treating CPSP is a big challenge for physicians. Management of CPSP includes both pharmacological and nonpharmacological treatment options. The analyses of the efficacy of pharmacological treatments in CPSP concluded that TCAs (amitriptyline) may have some beneficial effect. The effectiveness of antiepileptics (such as CBZ, gabapentin, lamotrigine, levetiracetam, or pregabalin) in the treatment of CPSP is still highly questionable. The above drugs can also be used in combinations. The role of opioids and anaesthetics in the management of CPSP is still under debate [64–67].

Nonpharmacological treatment in CPSP includes neurostimulatory techniques (such as motor cortex stimulation, deep brain stimulation (DBS), rTMS, and psychotherapy (e.g., CBT)). The data of these therapeutic approaches is still inconclusive due to the lack of well-designed RCTs [64–67]. Until now, the main limitation of motor cortex stimulation is the relatively low number of the treated patients. In addition, the efficacy of motor cortex stimulation depends on the accurate placement of the stimulation electrode. The findings regarding the effect of DBS in CPSP patients are also variable [64–67]. The results about the effectiveness of rTMS in CPSP are disappointing [64–67].

Overall, there is no clear evidence about the efficacy of either pharmacological or nonpharmacological therapeutic options in CPSP patients.

### 3.4. Chronic Central NP Associated with MS

The origin of NP in MS depends on the localization of the lesion in the somatosensory system. Pain related to spasticity in MS should be distinguished from NP, and it is a member of the subclass of musculoskeletal pain [4]. The prevalence of NP in MS is estimated to be around 23% [17]. There are no MS-specific recommendations for the pharmacological treatment of MS-associated chronic NP. Among the available medications, TCAs (e.g., amitriptyline), gabapentinoids (i.e., gabapentin and pregabalin), and SNRIs (e.g., venlafaxine and duloxetine) can be administered. In the case of MS-associated TN, CBZ or OXC is recommended [68, 69]. The medical use of cannabinoids (e.g., tetrahydrocannabinol (THC)/cannabidiol (CBD) oromucosal spray) in chronic central NP associated with MS might be beneficial, but generally, it is still a controversial issue due to their inconsistent results regarding their efficacy and numerous side effects [2, 12, 70, 71]. Regarding neuromodulation in MS-related NP, the following techniques seem to have promising effects: intrathecal baclofen, functional electrical stimulation, DBS, and spinal cord stimulation [72]. Regarding nonpharmacological interventions, including TENS, psychotherapy (e.g., telephone self-management or hypnosis), transcranial random noise stimulation, transcranial direct current stimulation (tDCS), hydrotherapy, and reflexology, there is at present a very low level of evidence to support their use in patients with chronic MS-related NP, based on a recent Cochrane meta-analysis [73].

## 4. Therapeutic Approaches for NP

Treatment options for NP can be divided into pharmacological and nonpharmacological (e.g., interventional, physical, and psychological therapies) approaches.

### 4.1. Pharmacological Therapeutic Options for NP

The therapeutic drug regimen for NP includes TCAs, SNRIs, antiepileptics, opioid analgesics, topical agents, and other drugs. We give the quality of evidence and the strength of recommendation of these pharmacons based on the Grading of Recommendation, Assessment, Development and Evaluation (GRADE) system [74] (Table 3).

#### 4.1.1. First-Line Pharmacological Treatments for NP

Among TCAs, amitriptyline (10-150 mg/day) is recommended as a first-line drug in the treatment of all NP conditions with strong recommendation based on moderate quality of evidence [2, 12, 13, 36, 74, 75]. Its major side effects are related to its anticholinergic effects (i.e., dry mouth, constipation, urinary retention, and orthostatic hypotension) [17, 36].

From the wide group of antiepileptics, gabapentinoids, such as gabapentin/gabapentin ER/enacarbil (1200-3600 mg/day tid) and pregabalin (300-600 mg/day bid), are the first-choice drugs in the treatment of all types of NP with strong recommendation based on high quality of evidence [2, 12, 13, 36, 74]. Regarding the latest Cochrane conclusion, the evidence for the efficacy of pregabalin in central NP is insufficient [31]. The major side effects of gabapentinoids are dizziness, sedation, and peripheral swelling [17, 36].

CBZ (200-400 mg/day) and OXC (300-600 mg/day) are recommended for TN [12, 76].

From the SNRI group, venlafaxine (150-225 mg/day once a day) and duloxetine (60-120 mg/day once a day) are the first-choice drugs with strong recommendation based on high quality of evidence for all NP conditions [2, 12, 13, 36, 74]. The most common side effect of SNRIs is nausea [17, 36].

#### 4.1.2. Second-Line Drug Treatments for NP

The opioid analgesics tramadol/tramadol ER (200-400 mg/day bid) and tapentadol (50-600 mg/day) are second-choice drugs with weak recommendation based on moderate quality of evidence for all types of NP [2, 12, 13, 36, 74]. The most common side effects of opioids are nausea, vomiting, and constipation [36].

Regarding topical agents, lidocaine (5%) plaster and capsaicin (8%) patch are recommended as second-choice drugs in the treatment of peripheral NP. Lidocaine patches have weak recommendation based on low quality of evidence, whereas capsaicin patches have weak recommendation based on high quality of evidence in the case of peripheral NP. [2, 12, 13, 36, 74]. The main side effects of these topical agents are erythema and itching [36].

#### 4.1.3. Third-Line Drug Treatments for NP

The strong opioids, morphine (10-120 mg/day) and oxycodone (10-120 mg/day), are recommended as third-line pharmacotherapeutic options with weak recommendation based on moderate quality of evidence for all NP conditions. The neurotoxin, BoNTA subcutaneously (50-200 IU BoNTA in 0.9% saline every three months), is a third-choice treatment option with weak recommendation based on low quality of evidence for peripheral NP [2, 12, 13, 36, 74]. The main side effect of BoNTA treatment is pain at injection site [36].

#### 4.1.4. Other Therapeutic Options in the Pharmacological Treatment of NP

The role of cannabis-based medicines (herbal cannabis, plant-derived, or synthetic THC or THC/CBD oromucosal spray) in chronic NP conditions has not yet been established, as their potential benefits and harms are not clear [77]. Oromucosal cannabinoids might have a beneficial effect in MS-related central NP and in peripheral NP with allodynia [2]. The effect of herbal medicinal products (e.g., nutmeg or St. John's wort) is still controversial according to a recent Cochrane review [78].

### 4.2. Nonpharmacological Therapeutic Options for NP

This category includes interventional, physical, and psychological therapies.

#### 4.2.1. Interventional Therapies for NP

Interventional treatments in different types of NP management include nerve blockades, epidural steroid injections, radiofrequency neuroablation, and intrathecal drug delivery as minimally invasive procedures, and peripheral and central neurostimulatory techniques (Table 4). Interventional treatments are indicated in intractable NP cases.

*(1) Peripheral Nerve Blockades*. The target of peripheral nerve blockades varies, depending on the affected peripheral nerves. The injected medications are local anaesthetics or their combination with opioids, clonidine, or steroids. The efficacy of peripheral nerve blockades in NP is still inconclusive [2, 79, 80].

*(2) Epidural Steroid Injection*. The efficacy of epidural corticosteroid (e.g., methylprednisolone, triamcinolone, betamethasone, or dexamethasone) injection for the treatment of painful radiculopathy is still debated, with the strength of recommendations ranging from weak to strong, and the quality of evidence is moderate [2, 79, 80]. Further studies are needed to clarify these inconsistent findings.

*(3) Sympathetic Nerve or Ganglion Treatments*. Sympathetic nerve or ganglion treatments can be performed by means of blockade, neurolysis, or ablation. The strength of recommendation of the sympathetic nerve block in the treatment of CRPS is inconclusive, and the quality of evidence is low. In truncal PHN, the strength of recommendation for sympathetic nerve blockades is against and the quality of evidence is moderate [2, 79–81].

*(4) Intrathecal Drug Delivery*. To date, only two medications (morphine and ziconotide) are applicable as intrathecal pain therapies for different types of chronic NP (e.g., truncal PHN, PDN, SCI, FBSS with radiculopathy, and CRPS). Well-designed clinical trials are still lacking. The strength of the recommendations in all of them is inconclusive, and the quality of evidences is low [2, 80, 82, 83].

*(5) Neurostimulation*. Neurostimulation is a nonpharmacological technique for the alleviation of NP. It can be divided into peripheral or central, and noninvasive or invasive neurostimulatory techniques. In the past years, several clinical studies have been published in this field. The diverse results of these studies have been evaluated by the GRADE system [2, 80, 82, 83].

- Peripheral Neurostimulation

- *Peripheral Nerve/Field Stimulation*. Peripheral nerve/field stimulation (subcutaneous) was effective in chronic and intractable low back pain [84–87]
- *TENS*. The Cochrane systematic analyses concluded that the quality of evidence regarding the usefulness of TENS in the treatment of NP is very low [87–89]
- *Dorsal root ganglion (DRG) stimulation*. A long-term, one-year outcome study revealed that DRG stimulation was effective in chronic NP; the pain was diminished by 56% at 12 months after the implantation of the leads [90]. A recent literature review has reported that the usefulness of DRG stimulation is supported by a high level of evidence. DRG stimulation was superior to spinal cord stimulation (SCS) in alleviating NP in CRPS and causalgia of the lower limb [87, 91]

(b) *Central Neurostimulation*. As regards the usefulness of central neurostimulatory techniques in intractable different NP conditions, weak recommendations could be established for SCS, epidural motor cortex stimulation, rTMS of primary motor cortex, and tDCS of primary motor cortex [87, 92]. Inconclusive results were found for DBS, rTMS of the dorsolateral prefrontal cortex, and tDCS of the dorsolateral prefrontal cortex in NP and for tDCS of the primary motor cortex in SCI-associated NP [87, 92]. SCS has been studied in different types of drug-refractory NP. Based on the GRADE classification, the strength of recommendations of SCS in truncal PHN, PDN, CRPS II, SCI-associated NP, and CPSP are inconclusive and quality of evidences are low. In FBSS with radiculopathy and CRPS I, the strength of recommendation for SCS is weak, and the quality of evidence is moderate [80]

#### 4.2.2. Physical Therapies for NP

Physical therapies are optional add-on possibilities, when pharmacological treatment options do not yield not satisfactory results.

There are numerous physical therapy modalities which can be applicable in NP, including the following: heat and cold applications, fluidotherapy, whirlpool, massage, ultrasound, short-wave diathermy, low-frequency currents (such as TENS, diadynamic currents, and interferential currents), high-voltage galvanic stimulation, and laser. These techniques have been investigated in different types of NP such as SCI, chronic postsurgical pain, radiculopathies, and PDP; however, the results are still inconclusive [93]. Regarding rehabilitation techniques of NP patients, relaxation techniques, acupuncture, mirror therapy, graded motor imagery, and visual illusion can be used in the management of different forms of NP, including SCI-associated NP, phantom pain, CRPS, and CPSP [2, 93]. Exercise training might be beneficial in the treatment of peripheral NP patients [94]. A systematic review revealed that exercise therapy combined with psychological therapy (such as mindfulness meditation, CBT, and mindfulness-based stress reduction), aerobic exercise (e.g., walking), and Thai Chi (as a strength-stability exercise) showed a moderate effect on the physical activity and quality of life in patients of PDP [95].

#### 4.2.3. Psychological Treatments for NP

One of the main aims of psychological treatments in chronic pain conditions (including chronic NP) is to diminish the intensity of pain, distress, and disability and to improve mood. CBT, but not behavioural therapy, has a weak effect in alleviating chronic pain; it has a small effect on disability; however, it is effective in improving mood and catastrophizing outcomes [2, 96]. Internet-delivered psychological therapies in nonheadache chronic pain patients showed a tendency to reduce pain, disability, depression, and anxiety. This new method showed a similar effect to that of the conventional face-to-face psychological intervention [97]. In chronic NP conditions in adults, well-designed clinical studies of psychological treatments are lacking. Two small clinical trials on CBT and psychotherapy demonstrated insufficient evidence concerning its efficacy and safety in chronic NP [98]. In burning mouth syndrome (BMS) with neuropathic component, a chronic primary pain condition, cognitive psychotherapy has a role in the management [99]. Based on the latest Cochrane systematic review evaluating the treatment in BMS, there was no RCTs assessing psychological therapies that evaluated short-term pain relief, whereas the evidence for the efficacy of psychological therapies in BMS patients to provide long-term symptom relief is of very low quality [100]. Hypnosis was given low grades of recommendations in the treatment of chronic phantom limb pain, SCI-related NP, and MS-associated NP. The recommendation of CBT in PDP and NP associated with cancer or HIV patients was graded as a good practice point (GPP) [101].

## 5. Conclusion

The peripheral and central NP conditions have high prevalence and have a deep impact on the quality of life of the patients. Alleviating this devastating pain condition is challenging for healthcare professionals. The novelty of this present review is the integration of the latest IASP classification of chronic pain with the International Classification of Diseases (ICD-11), first in the literature. Overviewing the last 10 years of relevant literature, we highlight that there are no specific drugs for the treatment of either peripheral or central NP. In this field, a major improvement is that the Neuropathic Pain Special Interest Group (NeuPSIG) of the IASP has developed a grading system in order to guide drug selection for the good clinical practice. A real breakthrough regarding nonpharmacological therapeutic options for NP conditions in the last decade is that clinical trials have been conducted, meta-analyses have been published, and guidelines have been released.

In the near future, the development of personalized and NP subtype-specific treatments are needed. In intractable NP cases, invasive nonpharmacological therapeutic options can be chosen; however, further high-quality clinical trials are necessary.

## Figures and Tables

**Table 1:** The IASP classification of chronic pain [35].

Chronic pain	
Chronic primary pain syndromes	Chronic secondary pain syndromes
Chronic widespread pain	Chronic cancer-related pain
Complex regional pain syndrome	Chronic postsurgical or posttraumatic pain
	Chronic NP
Chronic primary headache or orofacial pain	Chronic secondary headache or orofacial pain
Chronic primary visceral pain	Chronic secondary visceral pain
Chronic primary musculoskeletal pain	Chronic secondary musculoskeletal pain

Abbreviation: NP = neuropathic pain.

**Table 2:** The IASP classification of chronic NP [4].

Chronic neuropathic pain	
Chronic peripheral neuropathic pain	Chronic central neuropathic pain
Trigeminal neuralgia	Chronic central NP associated with spinal cord injury
Chronic NP after peripheral nerve injury	Chronic central NP associated with brain injury
Painful polyneuropathy	Chronic central poststroke pain
Postherpetic neuralgia	Chronic central NP associated with multiple sclerosis
Painful radiculopathy	

Abbreviation: NP = neuropathic pain.

**Table 3:** Pharmacological therapeutic options for neuropathic pain.

	Indications	Recommended dosage	Side effects	Comments	Ref.
First-line drugs					
TCAs	All types of NP	Amitriptyline: 10-150 mg/day	Dry mouth, constipation, urinary retention, orthostatic hypotension	Moderate quality of evidence; strong recommendation	[2, 12, 13, 36, 74]

Gabapentinoids	All types of NP	Gabapentin: 300-3600 mg/day Pregabalin: 150-600 mg/day	Dizziness, sedation, peripheral swelling	High quality of evidence; strong recommendation	[2, 12, 13, 36, 74]

SNRIs	All types of NP	Duloxetine: 20-120 mg/day Venlafaxine: 150-225 mg/day	Nausea	High quality of evidence; strong recommendation	[2, 12, 13, 36, 74]

Anticonvulsants (sodium ion channel blockers)	Trigeminal neuralgia	Carbamazepine: 200-400 mg/day Oxcarbazepine: 300-600 mg/day	Sedation, hepatotoxicity, hyponatraemia	GRADE recommendation is not applicable	[2, 12, 13, 36, 74]

Second-line drugs					
Weak opioids	All types of NP	Tramadol: 25-400 mg/day Tapentadol: 50-600 mg/day	Nausea, vomiting, constipation	Moderate quality of evidence; weak recommendation	[2, 12, 13, 36, 74]

Topical agents	Peripheral NP	Lidocaine (5%) plaster Capsaicin (8%) patch	Erythema, itching	Lidocaine (5%) plaster: low quality of evidence; weak recommendation; capsaicin (8%) patch: high quality of evidence; weak recommendation	[2, 12, 13, 36, 74]

Third-line drugs					
Strong opioids	All types of NP	Morphine: 10-120 mg/day Oxycodone: 10-120 mg/day	Nausea, vomiting, constipation	Moderate quality of evidence; weak recommendation	[2, 12, 13, 36, 74]

Neurotoxin	Peripheral NP	Botulinum toxin type A	Pain at injection site	Low quality of evidence; weak recommendation	[2, 12, 13, 36, 74]

Abbreviations: NP = neuropathic pain; SNRI = serotonin norepinephrine reuptake inhibitor; TCA = tricyclic antidepressant.

**Table 4:** Nonpharmacological therapeutic options for neuropathic pain.

	Indications	Comments	Ref.
Interventional therapies			
Nerve blockade	Drug-refractory NP	Local anaesthetics or combination with opioids, clonidine, or steroids; inconclusive recommendation	[2, 79, 80]

Epidural corticosteroid injection	Drug-refractory painful radiculopathy	Methylprednisolone, triamcinolone, betamethasone, dexamethasone; moderate quality of evidence; weak strength of recommendation	[2, 79, 80]

Sympathetic nerve/ganglion treatment	Intractable NP	Blockade, neurolysis, or neuroablation	[2, 79–81]

Intrathecal drug delivery	Drug-resistant NP	Morphine, ziconotide	[2, 80, 82, 83]

Peripheral nerve/field stimulation	Intractable low back pain	Subcutaneous application	[84–87]

Transcutaneous electrical nerve stimulation (TENS)	Intractable NP	Very low level of evidence	[87–89]

Dorsal root ganglion stimulation	Drug-refractory CRPS and causalgia of the lower limb	High level of evidence	[87, 91]

Spinal cord stimulation (SCS)	Drug-refractory painful diabetic neuropathy, truncal PHN, SCI-associated NP, CPSP, FBSS with radiculopathy, CRPS I and II	Weak recommendation	[80, 87, 92]

Epidural motor cortex stimulation	Intractable NP	Weak recommendation	[87, 92]

Repetitive transcranial magnetic stimulation (rTMS) of the primary motor cortex	Intractable NP	Weak recommendation	[87, 92]

Transcranial direct current stimulation (tDCS) of the primary motor cortex	Intractable NP	Weak recommendation	[87, 92]

Deep brain stimulation (DBS); repetitive transcranial magnetic stimulation (rTMS) of the dorsolateral prefrontal cortex; transcranial direct current stimulation (tDCS) of the dorsolateral prefrontal cortex	Intractable NP	Inconclusive	[87, 92]

Transcranial direct current stimulation (tDCS) of the primary motor cortex	Intractable spinal cord injury-associated NP	Inconclusive	[87, 92]

Physical therapies			
Heat and cold applications, fluidotherapy, whirlpool, massage, ultrasound, short-wave diathermy, low-frequency currents (e.g., TENS, diadynamic currents and interferential currents), high-voltage galvanic stimulation, laser	Spinal cord injury-associated NP, chronic postsurgical pain, painful radiculopathies, and painful diabetic neuropathy	Inconclusive	[93]

Rehabilitation techniques (relaxation techniques, acupuncture, mirror therapy, graded motor imagery, visual illusion)	Spinal cord injury-associated NP, phantom pain, CRPS, and chronic poststroke NP	Not well-established	[2, 93]

Exercise training	All types of NP	Beneficial effect	[94]

Exercise therapy combined with psychological therapy	Painful diabetic neuropathy	Moderate effect	[95]

Psychological therapies			
Cognitive behavioural therapy (CBT)	Chronic NP; painful diabetic neuropathy, cancer-associated NP, HIV-associated NP	Effective in improving mood and catastrophizing outcomes; good practice point	[2, 96, 101]

Internet-delivered psychological therapies	Nonheadache chronic pain	Similar effect to that of conventional face-to-face psychological intervention	[97]

Hypnosis	Chronic phantom limb pain, spinal cord injury-related NP, and multiple sclerosis-associated NP	Low level of evidence	[101]

Abbreviations: CRPS = complex regional pain syndrome; HIV = human immunodeficiency virus; NP = neuropathic pain.

## References

[1] Jensen T. S., Baron R., Haanpää M. (2011). A new definition of neuropathic pain. *Pain*.

[2] Colloca L., Ludman T., Bouhassira D. (2017). Neuropathic pain. *Nature Reviews Disease Primers*.

[3] Freynhagen R., Parada H. A., Calderon-Ospina C. A. (2019). Current understanding of the mixed pain concept: a brief narrative review. *Current Medical Research and Opinion*.

[4] Scholz J., Finnerup N. B., Attal N. (2019). The IASP classification of chronic pain for ICD-11: chronic neuropathic pain. *Pain*.

[5] Treede R. D. (2019). The role of quantitative sensory testing in the prediction of chronic pain. *Pain*.

[6] Arnold M. (2018). Headache Classification Committee of the International Headache Society (IHS) The International Classification of Headache Disorders, 3rd edition. *Cephalalgia*.

[7] Cruccu G., Finnerup N. B., Jensen T. S. (2016). Trigeminal neuralgia: new classification and diagnostic grading for practice and research. *Neurology*.

[8] Bendtsen L., Zakrzewska J. M., Abbott J. (2019). European Academy of Neurology guideline on trigeminal neuralgia. *European Journal of Neurology*.

[9] Shaefer J. R., Khawaja S. N., Bavia P. F. (2018). Sex, gender, and orofacial pain. *Dental Clinics of North America*.

[10] Benoliel R., Svensson P., Evers S. (2019). The IASP classification of chronic pain for ICD-11: chronic secondary headache or orofacial pain. *Pain*.

[11] Maarbjerg S., Di Stefano G., Bendtsen L., Cruccu G. (2017). Trigeminal neuralgia - diagnosis and treatment. *Cephalalgia*.

[12] Attal N., Cruccu G., Baron R. (2010). EFNS guidelines on the pharmacological treatment of neuropathic pain: 2010 revision. *European Journal of Neurology*.

[13] Moulin D., Boulanger A., Clark A. J. (2014). Pharmacological management of chronic neuropathic pain: revised consensus statement from the Canadian Pain Society. *Pain Research & Management*.

[14] Di Stefano G., Truini A., Cruccu G. (2018). Current and innovative pharmacological options to treat typical and atypical trigeminal neuralgia. *Drugs*.

[15] Schug S. A., Lavand’homme P., Barke A., Korwisi B., Rief W., Treede R.-D. (2019). The IASP classification of chronic pain for ICD-11. *Pain*.

[16] Nikolajsen L., Ilkjaer S., Christensen J. H., Kroner K., Jensen T. S. (1998). Pain after amputation. *British Journal of Anaesthesia*.

[17] Kerstman E., Ahn S., Battu S., Tariq S., Grabois M. (2013). Neuropathic pain. *Handbook of Clinical Neurology*.

[18] MJM A., Hale T., Dungca M. (2011). Pharmacologic interventions for treating phantom limb pain. *The Cochrane Database of Systematic Reviews*.

[19] Wylde V., Dennis J., Beswick A. D. (2017). Systematic review of management of chronic pain after surgery. *The British Journal of Surgery*.

[20] Cho J. H., Lee J. H., Song K. S., Hong J. Y. (2017). Neuropathic pain after spinal surgery. *Asian Spine Journal*.

[21] Nicholls J. L., Azam M. A., Burns L. C. (2018). Psychological treatments for the management of postsurgical pain: a systematic review of randomized controlled trials. *Patient Related Outcome Measures*.

[22] Bottiger B. A., Esper S. A., Stafford-Smith M. (2014). Pain management strategies for thoracotomy and thoracic pain syndromes. *Seminars in Cardiothoracic and Vascular Anesthesia*.

[23] Humble S. R., Dalton A. J., Li L. (2015). A systematic review of therapeutic interventions to reduce acute and chronic post-surgical pain after amputation, thoracotomy or mastectomy. *European Journal of Pain*.

[24] Larsson I. M., Ahm Sorensen J., Bille C. (2017). The post-mastectomy pain syndrome-a systematic review of the treatment modalities. *The Breast Journal*.

[25] Werner M. U., Bischoff J. M., Rathmell J. P., Kehlet H. (2012). Pulsed radiofrequency in the treatment of persistent pain after inguinal herniotomy: a systematic review. *Regional Anesthesia and Pain Medicine*.

[26] Brandsborg B., Nikolajsen L. (2018). Chronic pain after hysterectomy. *Current Opinion in Anaesthesiology*.

[27] Blanton E., Lamvu G., Patanwala I. (2017). Non-opioid pain management in benign minimally invasive hysterectomy: a systematic review. *American Journal of Obstetrics and Gynecology*.

[28] Lirk P., Thiry J., Bonnet M. P., Joshi G. P., Bonnet F., PROSPECT Working Group (2019). Pain management after laparoscopic hysterectomy: systematic review of literature and PROSPECT recommendations. *Regional Anesthesia and Pain Medicine*.

[29] Grosu I., Lavand’homme P., Thienpont E. (2014). Pain after knee arthroplasty: an unresolved issue. *Knee Surgery, Sports Traumatology, Arthroscopy*.

[30] Sakellariou V. I., Poultsides L. A., Ma Y., Bae J., Liu S., Sculco T. P. (2016). Risk assessment for chronic pain and patient satisfaction after total knee arthroplasty. *Orthopedics*.

[31] Derry S., Bell R. F., Straube S. (2019). Pregabalin for neuropathic pain in adults. *The Cochrane Database of Systematic Reviews*.

[32] Summer G. J., Puntillo K. A., Miaskowski C., Green P. G., Levine J. D. (2007). Burn injury pain: the continuing challenge. *The Journal of Pain*.

[33] Kneib C. J., Sibbett S. H., Carrougher G. J., Muffley L. A., Gibran N. S., Mandell S. P. (2019). The effects of early neuropathic pain control with gabapentin on long-term chronic pain and itch in burn patients. *Journal of Burn Care & Research*.

[34] Mewa Kinoo S., Singh B. (2017). Complex regional pain syndrome in burn pathological scarring: a case report and review of the literature. *Burns*.

[35] Treede R. D., Rief W., Barke A. (2019). Chronic pain as a symptom or a disease: the IASP Classification of Chronic Pain for the International Classification of Diseases (ICD-11). *Pain*.

[36] Cavalli E., Mammana S., Nicoletti F., Bramanti P., Mazzon E. (2019). The neuropathic pain: an overview of the current treatment and future therapeutic approaches. *International Journal of Immunopathology and Pharmacology*.

[37] Stevanato G., Devigili G., Eleopra R. (2014). Chronic post-traumatic neuropathic pain of brachial plexus and upper limb: a new technique of peripheral nerve stimulation. *Neurosurgical Review*.

[38] Bouhassira D. (2019). Neuropathic pain: definition, assessment and epidemiology. *Revue Neurologique*.

[39] Tajti J., Szok D., Majlath Z., Csati A., Petrovics-Balog A., Vecsei L. (2016). Alleviation of pain in painful diabetic neuropathy. *Expert Opinion on Drug Metabolism & Toxicology*.

[40] Iqbal Z., Azmi S., Yadav R. (2018). Diabetic peripheral neuropathy: epidemiology, diagnosis, and pharmacotherapy. *Clinical Therapeutics*.

[41] Wiffen P. J., Derry S., Bell R. F. (2017). Gabapentin for chronic neuropathic pain in adults. *The Cochrane Database of Systematic Reviews*.

[42] MPT L., RAC H., Wiffen P. J. (2014). Duloxetine for treating painful neuropathy, chronic pain or fibromyalgia. *The Cochrane Database of Systematic Reviews*.

[43] Zhou M., Chen N., He L., Yang M., Zhu C., Wu F. (2017). Oxcarbazepine for neuropathic pain. *The Cochrane Database of Systematic Reviews*.

[44] Gaskell H., Derry S., Stannard C., Moore R. A. (2016). Oxycodone for neuropathic pain in adults. *The Cochrane Database of Systematic Reviews*.

[45] Derry S., Rice A. S., Cole P., Tan T., Moore R. A. (2017). Topical capsaicin (high concentration) for chronic neuropathic pain in adults. *The Cochrane Database of Systematic Reviews*.

[46] Schutzer-Weissmann J., Farquhar-Smith P. (2017). Post-herpetic neuralgia - a review of current management and future directions. *Expert Opinion on Pharmacotherapy*.

[47] Romano C. L., Romano D., Bonora C., Mineo G. (2009). Pregabalin, celecoxib, and their combination for treatment of chronic low-back pain. *Journal of Orthopaedics and Traumatology*.

[48] Freynhagen R., Baron R. (2009). The evaluation of neuropathic components in low back pain. *Current Pain and Headache Reports*.

[49] Andrasinova T., Kalikova E., Kopacik R. (2019). Evaluation of the neuropathic component of chronic low back pain. *The Clinical Journal of Pain*.

[50] Saldana M. T., Navarro A., Perez C., Masramon X., Rejas J. (2010). Patient-reported-outcomes in subjects with painful lumbar or cervical radiculopathy treated with pregabalin: evidence from medical practice in primary care settings. *Rheumatology International*.

[51] Mathieson S., Maher C. G., McLachlan A. J. (2017). Trial of pregabalin for acute and chronic sciatica. *The New England Journal of Medicine*.

[52] Patel E. A., Perloff M. D. (2018). Radicular pain syndromes: cervical, lumbar, and spinal stenosis. *Seminars in Neurology*.

[53] Urits I., Burshtein A., Sharma M. (2019). Low back pain, a comprehensive review: pathophysiology, diagnosis, and treatment. *Current Pain and Headache Reports*.

[54] Park J., Chung M. E. (2018). Botulinum toxin for central neuropathic pain. *Toxins*.

[55] Han Z. A., Song D. H., Oh H. M., Chung M. E. (2016). Botulinum toxin type a for neuropathic pain in patients with spinal cord injury. *Annals of Neurology*.

[56] Boldt I., Eriks-Hoogland I., Brinkhof M. W., de Bie R., Joggi D., von Elm E. (2014). Non-pharmacological interventions for chronic pain in people with spinal cord injury. *The Cochrane Database of Systematic Reviews*.

[57] Cassidy J. D., Carroll L. J., Peloso P. M. (2004). Incidence, risk factors and prevention of mild traumatic brain injury: results of the WHO Collaborating Centre Task Force on Mild Traumatic Brain Injury. *Journal of Rehabilitation Medicine*.

[58] Irvine K. A., Clark J. D. (2018). Chronic pain after traumatic brain injury: pathophysiology and pain mechanisms. *Pain Medicine*.

[59] Stocchetti N., Carbonara M., Citerio G. (2017). Severe traumatic brain injury: targeted management in the intensive care unit. *The Lancet Neurology*.

[60] Grandhi R., Tavakoli S., Ortega C., Simmonds M. J. (2017). A review of chronic pain and cognitive, mood, and motor dysfunction following mild traumatic brain injury: complex, comorbid, and/or overlapping conditions?. *Brain Sciences*.

[61] Gironda R. J., Clark M. E., Ruff R. L. (2009). Traumatic brain injury, polytrauma, and pain: challenges and treatment strategies for the polytrauma rehabilitation. *Rehabilitation Psychology*.

[62] Choi G. S., Kwak S. G., Lee H. D., Chang M. C. (2018). Effect of high-frequency repetitive transcranial magnetic stimulation on chronic central pain after mild traumatic brain injury: a pilot study. *Journal of Rehabilitation Medicine*.

[63] Khoury S., Benavides R. (2018). Pain with traumatic brain injury and psychological disorders. *Progress in Neuro-psychopharmacology & Biological Psychiatry*.

[64] Harrison R. A., Field T. S. (2015). Post stroke pain: identification, assessment, and therapy. *Cerebrovascular Diseases*.

[65] Oh H., Seo W. (2015). A comprehensive review of central post-stroke pain. *Pain Management Nursing*.

[66] Delpont B., Blanc C., Osseby G. V., Hervieu-Begue M., Giroud M., Bejot Y. (2018). Pain after stroke: a review. *Revue Neurologique*.

[67] Kim J. S. (2014). Pharmacological management of central post-stroke pain: a practical guide. *CNS Drugs*.

[68] Duffy S. S., Lees J. G., Perera C. J., Moalem-Taylor G. (2018). Managing neuropathic pain in multiple sclerosis: pharmacological interventions. *Medicinal Chemistry*.

[69] Murphy K. L., Bethea J. R., Fischer R., Zagon I. S., McLaughlin P. J. (2017). Neuropathic pain in multiple sclerosis-current therapeutic intervention and future treatment perspectives. *Multiple Sclerosis: Perspectives in Treatment and Pathogenesis*.

[70] Fraguas-Sanchez A. I., Torres-Suarez A. I. (2018). Medical use of cannabinoids. *Drugs*.

[71] Rice J., Cameron M. (2018). Cannabinoids for treatment of MS symptoms: state of the evidence. *Current Neurology and Neuroscience Reports*.

[72] Abboud H., Hill E., Siddiqui J., Serra A., Walter B. (2017). Neuromodulation in multiple sclerosis. *Multiple Sclerosis*.

[73] Amatya B., Young J., Khan F. (2018). Non-pharmacological interventions for chronic pain in multiple sclerosis. *The Cochrane Database of Systematic Reviews*.

[74] Finnerup N. B., Attal N., Haroutounian S. (2015). Pharmacotherapy for neuropathic pain in adults: a systematic review and meta-analysis. *The Lancet Neurology*.

[75] Beniczky S., Tajti J., Timea Varga E., Vecsei L. (2005). Evidence-based pharmacological treatment of neuropathic pain syndromes. *Journal of Neural Transmission*.

[76] Cruccu G., Truini A. (2017). A review of neuropathic pain: from guidelines to clinical practice. *Pain and therapy*.

[77] Mucke M., Phillips T., Radbruch L., Petzke F., Hauser W. (2018). Cannabis-based medicines for chronic neuropathic pain in adults. *The Cochrane Database of Systematic Reviews*.

[78] Boyd A., Bleakley C., Hurley D. A. (2019). Herbal medicinal products or preparations for neuropathic pain. *The Cochrane Database of Systematic Reviews*.

[79] Sdrulla A., Chen G. (2016). Minimally invasive procedures for neuropathic pain. *Pain Management*.

[80] Dworkin R. H., O'Connor A. B., Kent J. (2013). Interventional management of neuropathic pain: NeuPSIG recommendations. *Pain*.

[81] Abramov R. (2014). Lumbar sympathetic treatment in the management of lower limb pain. *Current Pain and Headache Reports*.

[82] Prusik J., Argoff C., Peng S., Pilitsis J. G. (2017). Use of low dose ziconotide as first-line intrathecal monotherapy. *Neuromodulation*.

[83] Sukul V. V. (2019). Intrathecal pain therapy for the management of chronic noncancer pain. *Neurosurgery Clinics of North America*.

[84] McRoberts W. P., Wolkowitz R., Meyer D. J. (2013). Peripheral nerve field stimulation for the management of localized chronic intractable back pain: results from a randomized controlled study. *Neuromodulation*.

[85] Kloimstein H., Likar R., Kern M. (2014). Peripheral nerve field stimulation (PNFS) in chronic low back pain: a prospective multicenter study. *Neuromodulation*.

[86] Petersen E. A., Slavin K. V. (2014). Peripheral nerve/field stimulation for chronic pain. *Neurosurgery Clinics of North America*.

[87] Cruccu G., Aziz T. Z., Garcia-Larrea L. (2007). EFNS guidelines on neurostimulation therapy for neuropathic pain. *European Journal of Neurology*.

[88] Gibson W., Wand B. M., O'Connell N. E. (2017). Transcutaneous electrical nerve stimulation (TENS) for neuropathic pain in adults. *The Cochrane Database of Systematic Reviews*.

[89] Gibson W., Wand B. M., Meads C., Catley M. J., O'Connell N. E. (2019). Transcutaneous electrical nerve stimulation (TENS) for chronic pain - an overview of Cochrane reviews. *The Cochrane Database of Systematic Reviews*.

[90] Liem L., Russo M., Huygen F. J. P. M. (2015). One-year outcomes of spinal cord stimulation of the dorsal root ganglion in the treatment of chronic neuropathic pain. *Neuromodulation*.

[91] Harrison C., Epton S., Bojanic S., Green A. L., FitzGerald J. J. (2018). The efficacy and safety of dorsal root ganglion stimulation as a treatment for neuropathic pain: a literature review. *Neuromodulation*.

[92] Cruccu G., Garcia-Larrea L., Hansson P. (2016). EAN guidelines on central neurostimulation therapy in chronic pain conditions. *European Journal of Neurology*.

[93] Akyuz G., Kenis O. (2014). Physical therapy modalities and rehabilitation techniques in the management of neuropathic pain. *American Journal of Physical Medicine & Rehabilitation*.

[94] Dobson J. L., McMillan J., Li L. (2014). Benefits of exercise intervention in reducing neuropathic pain. *Frontiers in Cellular Neuroscience*.

[95] van Laake-Geelen C. C. M., Smeets R., Quadflieg S., Kleijnen J., Verbunt J. A. (2019). The effect of exercise therapy combined with psychological therapy on physical activity and quality of life in patients with painful diabetic neuropathy: a systematic review. *Scandinavian Journal of Pain*.

[96] Williams A. C., Eccleston C., Morley S. (2012). Psychological therapies for the management of chronic pain (excluding headache) in adults. *The Cochrane Database of Systematic Reviews*.

[97] Eccleston C., Fisher E., Craig L., Duggan G. B., Rosser B. A., Keogh E. (2014). Psychological therapies (internet-delivered) for the management of chronic pain in adults. *The Cochrane Database of Systematic Reviews*.

[98] Eccleston C., Hearn L., Williams A. C. (2015). Psychological therapies for the management of chronic neuropathic pain in adults. *The Cochrane Database of Systematic Reviews*.

[99] Kim M. J., Kho H. S. (2018). Understanding of burning mouth syndrome based on psychological aspects. *The Chinese Journal of Dental Research*.

[100] McMillan R., Forssell H., Buchanan J. A., Glenny A. M., Weldon J. C., Zakrzewska J. M. (2016). Interventions for treating burning mouth syndrome. *The Cochrane Database of Systematic Reviews*.

[101] Castelnuovo G., Giusti E. M., Manzoni G. M. (2016). Psychological treatments and psychotherapies in the neurorehabilitation of pain: evidences and recommendations from the Italian Consensus Conference on Pain in Neurorehabilitation. *Frontiers in Psychology*.

